# Role of Chemotherapy in Vulvar Cancers: Time to Rethink Standard of Care?

**DOI:** 10.3390/cancers13164061

**Published:** 2021-08-12

**Authors:** Marco Mazzotta, Laura Pizzuti, Eriseld Krasniqi, Francesca Sofia Di Lisa, Federico Cappuzzo, Lorenza Landi, Domenico Sergi, Fabio Pelle, Sonia Cappelli, Claudio Botti, Enrico Vizza, Silverio Tomao, Luca Marchetti, Giuseppe Sanguineti, Andrea Botticelli, Paolo Marchetti, Valentina Magri, Simona Pisegna, Aldo Venuti, Federica Tomao, Federica Buzzacchino, Gennaro Ciliberto, Maddalena Barba, Patrizia Vici

**Affiliations:** 1 Medical Oncology Unit, Belcolle Hospital, 01100 Viterbo, Italy; marcomazzotta88@gmail.com; 2 Division of Medical Oncology 2, IRCCS Regina Elena National Cancer Institute, 00144 Rome, Italy; laura.pizzuti@ifo.gov.it (L.P.); f.cappuzzo@gmail.com (F.C.); landi.lorenza@gmail.com (L.L.); domenico.sergi@ifo.gov.it (D.S.); 3 Medical Oncology A, Department of Radiological, Oncological and Anatomo-Pathological Sciences, Umberto I University Hospital, University Sapienza, 00185 Rome, Italy; fs.dilisa@libero.it (F.S.D.L.); silverio.tomao@gmail.com (S.T.); 4 Breast Surgery Unit, IRCCS Regina Elena National Cancer Institute, 00144 Rome, Italy; fabio.pelle@ifo.gov.it (F.P.); sonia.cappelli@ifo.gov.it (S.C.); claudio.botti@ifo.gov.it (C.B.); 5 Gynecologic Oncology Unit, IRCCS-Regina Elena National Cancer Institute, 00144 Rome, Italy; enrico.vizza@ifo.gov.it; 6 Division of Medical Oncology, Fatebenefratelli San Pietro Hospital, 00189 Rome, Italy; lucmarchetti@yahoo.it; 7 Radiotherapy Department, IRCCS Regina Elena National Cancer Institute, 00144 Rome, Italy; giuseppe.sanguineti@ifo.gov.it; 8 Medical Oncology B, Department of Radiological, Oncological and Anatomo-Pathological Sciences, Policlinico Umberto I, Sapienza University, 00185 Rome, Italy; andreabotticelli@hotmail.it (A.B.); paolo.marchetti@hotmail.it (P.M.); valentina.magri@uniroma1.it (V.M.); simona.pisegna@uniroma1.it (S.P.); 9 HPV-UNIT-UOSD Tumor Immunology and Immunotherapy, IRCCS Regina Elena National Cancer Institute, 00144 Rome, Italy; Aldo.venuti@ifo.gov.it; 10 Department of Gynecologic Oncology, European Institute of Oncology, IRCCS, 20141 Milan, Italy; federica.tomao@ieo.it; 11 Oncology Division, S. Giovanni Hospital, 00184 Rome, Italy; federica.buzzacchino@gmail.com; 12 Scientific Direction, IRCCS Regina Elena National Cancer Institute, 00144 Rome, Italy; gennaro.ciliberto@ifo.gov.it; 13 Sperimentazioni di Fase IV, IRCCS Regina Elena National Cancer Institute, 00144 Rome, Italy; patrizia.vici@ifo.gov.it

**Keywords:** vulvar cancer, chemotherapy, gynecologic cancer

## Abstract

**Simple Summary:**

Vulvar cancer is a difficult clinical condition to treat. Although it is not one of the most frequently diagnosed cancers, its incidence is not negligible. Treatment depends on the extent of the disease and is currently based on surgery, radiotherapy and chemotherapy. The combination of these possible treatments, in the context of multidisciplinary discussions, is crucial. In this paper we present a review of the data available in the literature on the role of chemotherapy in the treatment of vulvar cancer, with a look at future perspectives.

**Abstract:**

The actual role of chemotherapy in vulvar cancer is undeniably a niche topic. The low incidence of the disease limits the feasibility of randomized trials. Decision making is thus oriented by clinical and pathological features, whose relevance is generally weighted against evidence from observational studies and clinical practice. The therapeutic management of vulvar cancer is increasingly codified and refined at an individual patient level. It is of note that the attitude towards evidence sharing and discussion within a multidisciplinary frame is progressively consolidating. Viable options included in the therapeutic armamentarium available for vulvar cancer patients are frequently an adaption from standards used for cervical or anal carcinoma. Chemotherapy is more frequently combined with radiotherapy as neo-/adjuvant or definitive treatment. Drugs commonly used are platinum derivative, 5-fluorouracil and mitomicin C, mostly in combination with radiotherapy for radiosensitization. Exclusive chemotherapy in the neo-/adjuvant setting comprises platinum-derivative, combined with bleomicin and methotrexate, 5-fluorouracil, ifosfamide or taxanes. In advanced disease, current regimens include cisplatin-based chemoradiation, with or without 5-fluorouracil, or doublets with platinum in combination with a taxane. Our work is also enriched by a concise excursus on the biologic pathways underlying vulvar cancer. Introductory hints are also provided on targeted agents, a rapidly evolving research field.

## 1. Introduction

The incidence rate of vulvar cancers is set at about two to three newly diagnosed cases out of 100,000 women per year. According to the Global Cancer Statistics 2020, this translates into incidence and mortality estimates of 45,240 (age-standardized rate per 100,000 women–years: 0.9) and 17,427 (age-standardized rate per 100,000 women–years: 0.3) cases, respectively [1]. Among the gynecological cancers, vulvar cancers account for 2–5% of all diagnosed cases, with the highest rates present in older women and a median age at diagnosis of 68 years [2]. The still remarkable proportion of cases in an advanced stage at diagnosis makes vulvar cancer a clinical challenge for oncologists. 

In recent years, the HPV-related subtype of vulvar carcinomas has become increasingly common. The role of HPV infection in cervical cancers is widely known. Indeed, HPV can be linked to other types of malignancies, e.g., anogenital cancer (vulvar, vaginal, anal and penial), head and neck cancers (HNCs) and genital warts in both genders [3]. Overall, HPV accounts for about 40–50% of vaginal and vulvar cancers and more than 60% of penile cancers. 

**Figure 1:**
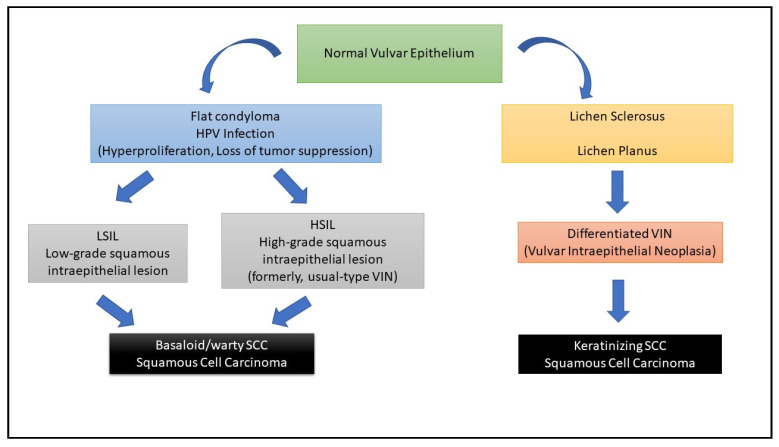
Schematic representation of the pathophysiology of the vulvar cancer.

From a histological standpoint, vulvar cancers are most commonly squamous cell carcinomas (SCC) related to HPV infection, particularly to the HPV 16 subtype [4]. HPV-related vulvar carcinomas mostly arise in younger women. The introduction of HPV vaccination is expected to partially reduce the incidence of squamous vulvar cancers within the next twenty years [5]. Alternative pathways involved in vulvar cancer pathogenesis arise from chronic dermatoses as lichen sclerosus or planus [6]. Less frequent histologies, in about 10% of cases, are basal cell carcinoma, melanoma, Paget’s disease, Bartholin gland adenocarcinoma, neuroendocrine tumors and sarcomas. The early identification of premalignant lesions, such as differentiated vulvar intraepithelial neoplasia (VIN) (from low-grade to high-grade), lichen sclerosus, and lichen planus, is of pivotal importance to effectively reduce the occurrence of invasive vulvar cancer [7,8,9], (Figure 1). 

Clinical management of vulvar cancer patients is defined upon several factors, including histology and stage, but also age, comorbidities and performance status at diagnosis. Treatment decisions should be taken at an individual patient level by a multidisciplinary team. Radical surgery in early stages, adjuvant radiotherapy in the case of node involvement or positive resection margins and chemoradiation for more advanced stages represent some of the basic tenets of current treatment orientations [8]. In locally advanced vulvar cancer, upfront surgery lacks appropriate indication. In these patients, the combination of chemoradiation may be the preferred choice. Overall, the administration of neoadjuvant chemotherapy remains a not negligible option. In the metastatic setting, the main focus is set on palliative care and quality of life. To this purpose, chemoradiation or systemic chemotherapy or, more recently, their combination with immunotherapy or biologic agents, represent valuable options [9]. In the work herein presented, we electively focused on the use of systemic chemotherapy in vulvar cancers across the different settings. Overall, there are no substantial doubts in stating that the experience matured thus far is detrimental and the results obtained quite disappointing. Our efforts are thus centered on the critical interpretation of the available literature, particularly in light of the objective limitations to be overcome in future studies. Indeed, the available manuscripts mostly include data from case-series including a limited number of patients. For the vast majority, these works date back to decades ago and the evidence reported relates to “old” therapeutic agents [10]. Beyond the punctual analysis of relevant aspects emerging at the single study level, anticipated remarks of some common traits of the below reported literature may help critic interpretation of the available evidence. First, the vast majority of these studies are limited in size. This imposes limits to the generalizability of the results obtained. To at least partly minimize the magnitude of this phenomenon, a minimum sample size of 10 patients was a prerequisite for inclusion in our review. However, we also considered studies with lower numbers when paucity of data was a particularly relevant issue. Second, the regimens employed as radiosensitizing agents are considerably heterogeneous, with cisplatin, followed by cisplatin combined with 5-fluorouracil and 5-fluorouracil combined with mitomycin C being the most commonly used. A further note concerning toxicity: split courses of radiotherapy, with an interval of 14 days, have become increasingly used since associated less commonly with severe toxicity. However, data reporting on toxicity, as well as on quality of life, often lacks or is incomplete.

In addition, the use of these agents is discussed in reference to recent advances in knowledge on the etiopathogenesis of vulvar cancer and related underlying mechanisms in an attempt to depict new therapeutic scenarios based on the use of targeted agents.

## 2. Early Setting

Surgery is the mainstay of treatment for the vast majority of patients with vulvar cancer. It possibly includes radical local excision or radical vulvectomy with bilateral inguinal lymphadenectomy or sentinel node dissection in patients with not-bulky disease [11]. In the case of nodal involvement and/or positive resection margins with no chance for re-excision, postoperative adjuvant inguinal and pelvic radiotherapy are appropriate. Doses vary within a 50 to 60 Gray (Gy) range depending on the amount of metastatic spread [12]. 

The degree of therapeutic aggressiveness increases with the extent of locoregional involvement. In more advanced stages, surgery may be extended to involve the anus, urethra and vagina, up to pelvic exenteratio. In these patients, systemic therapy with a neoadjuvant intent or, even more frequently, chemoradiation, is a viable option. Surgery follows, if its feasibility is verified [13]. Preoperative radiotherapy is a reasonable alternative to pelvic exenteratio, having shown the ability to effectively reduce tumor burden and the extent of subsequent surgery [14].

Currently, chemoradiation is considered the gold standard in III/IVA stages. Indeed, in patients with locally advanced disease, its use is associated with significantly improved response rate and overall survival compared to radiotherapy alone [15]. In addition, in patients not amenable to surgery, definitive chemoradiation showed more favorable survival outcomes compared to definitive radiation alone [16].

The addition of adjuvant chemotherapy in combination with radiotherapy in node-positive disease, compared to radiotherapy alone, has demonstrated a clear advantage in reduction of risk of death, as reported by the National Cancer Database analysis. In more detail, according to the results of adjusted Cox proportional regression models including data from 1797 patients, the outcome analysis translated into a 38% risk reduction in patients treated with adjuvant chemotherapy (*p* < 0.001) [17]. 

### 2.1. Neoadjuvant Chemoradiation

Consistent evidence supports the use of chemoradiation as neoadjuvant treatment in locally advanced vulvar carcinoma followed by surgery. The inherent studies have been listed in Table 1. 

Chemoradiation in locally advanced vulvar cancer was first used by Thomas and colleagues [18]. These authors reported on the use of 5-fluorouracil and mitomicin C combined with radiotherapy in 9 patients out of a larger series of 33. Six patients from this subgroup achieved a complete response. The use of 5-fluorouracil and cisplatin combined with radiotherapy in 12 locally advanced vulvar cancer patients resulted in a 67% clinical complete response rate [19]. Ten patients were free from recurrence after a median follow up of 37 months. The use of this same regimen by Eifel and coauthors in 11 primary vulvar cancer patients resulted into a 100% clinical partial response rate. Of eight patients who underwent vulvar resection 6 weeks after chemoradiation, a complete pathological response was achieved in four patients. Two-year overall survival was set at 58% [20]. Scheistroen and colleagues carried out a trial involving forty-two patients with advanced squamous cell carcinoma of the vulva that were treated with a combination regimen of bleomycin 180 mg and external irradiation of 30–45 Gy [21]. Of the twenty patients with primary lesions, fifteen (75%) showed objective response (five showing complete and ten showing partial response). The combined use of 5-fluorouracil with mitomicin C in 24 vulvar cancer patients with locally advanced disease translated into a clinical response of 91.6% [22]. In the study from Landoni and coauthors, 58 patients received chemoradiation with 5-fluorouracil and mitomycin C, observing a pathological complete response in both vulva and nodes in 24.4% of 41 patients with locally advanced tumors and in 17.6% of the 17 patients with recurred tumors. The 2-year overall survival was 36% [23].

A further study reported on the outcome of radiotherapy and 5-fluorouracil alone or in combination with cisplatin in 23 vulvar carcinoma patients. In 14 patients (60.9%), a clinical complete response was recorded. Fourteen patients were free of recurrence at 42 months of median follow-up [24]. The GOG 101 study addressed the topic of concurrent radiotherapy and cisplatin/infusional 5-fluorouracil in 71 patients with unresectable vulvar cancer [25]. The authors observed a clinical complete response of 48%. In 31 patients who underwent surgery, there was a 70% pathological complete response rate. Toxicity was moderate. At a median follow up of 50 months, 43 of 71 patients with “unresectable” disease showed no evidence of disease. The prospective GOG study aimed at evaluating the outcomes of 46 patients diagnosed with squamous vulvar carcinoma and N2/N3 nodal involvement [26]. Patients participating in this trial were exposed to a split course of radiation, 4760 cGy to the primary and lymph nodes, with concurrent chemotherapy, cisplatin/5-FU, followed by surgery. Overall, 38 out of 46 patients received subsequent surgery, with a pathological complete response of 40% in the nodes and 52% in the vulva tumor bed. At a median follow up of 78 months, 32% of the patients showed no evidence of recurrence. Han and coauthors treated 14 patients with radiotherapy combined with 5-fluorouracil and mitomicin C or cisplatin. The authors reported a 71.4% complete clinical response rate [27].

To our knowledge, two systematic reviews addressed the topic of neoadjuvant chemoradiation for advanced primary vulvar cancer [32,33]. In the work from Van Doom and colleagues, the systematic search of the literature yielded no results for randomized clinical trials. The five studies that met the inclusion criteria were included in the present work and are listed in Table 1 and Table 2. When globally considered, evidence from the studies included support for the role of preoperative chemoradiotherapy in reducing tumor size and improving surgical outcomes. Notably, in a considerable proportion of patients, locoregional adverse events complicate the clinical course following the therapeutic approach under consideration. In addition, no evidence on the effects on quality of life (QOL) was available. The substantial degree of heterogeneity did not allow for a quantitative data synthesis, i.e., no meta-analysis was performed [32]. 

A further Cochrane systemic review on chemoradiation in vulvar carcinoma was published in 2011. Based on the evidence reviewed, no significant difference in overall survival or treatment-related adverse events emerged when comparing chemoradiation with primary surgery. These results need careful interpretation in light of the following key points. Only three trials met the preset eligibility criteria and were thus included. Only one of these was a randomized clinical trial, while the two remaining were retrospective in nature. The review is based on data on 141 women only. The remarkable heterogeneity among the studies deemed eligible for inclusion made the meta-analysis unfeasible. The authors concluded that the paucity and overall low quality of evidence made the drawing any satisfactory conclusions concerning the original research question impossible [33]. The only randomized trial included in the previously cited Cochrane review is from Maneo and collaborators. This work showed evidence on outcomes of neoadjuvant chemoradiation compared with primary surgery followed by radiotherapy in locally advanced vulvar cancer. Administered chemotherapy was infusional 5-fluorouracil and mitomycin C. No significant differences in the risk of death between the two treatment modalities emerged [34]. Gertzen and coauthors reported on the outcomes of a fractionated schedule of radiotherapy delivered twice daily combined with 5-fluorouracil and cisplatin in 18 patients. A clinical complete response was reported in 72.2% of patients [28].

Gaudineau and collaborators administered radiotherapy and different cisplatinum-based chemotherapy regimens in 22 patients. The authors described a 27% of pathological complete response and 95% of pathological partial response following neoadjuvant therapy [29].

In the phase II GOG 205 trial, Moore and coauthors treated 58 patients with T3/T4 vulvar carcinoma with radiotherapy concomitant with cisplatin. In 37 patients, the authors observed a clinical response, while a pathological complete response was recorded in 50% of cancer patients. At a median follow up of 24 months, 31 patients showed no evidence of recurrence [30]. Beriwal and colleagues reported quite a large experience on 42 patients treated with chemotherapy and intensity modulated radiation therapy (IMRT), with good pathologic response and acceptable toxicities [31].

A meta-analysis on chemoradiation in elderly and nonelderly patients included data from seven studies for a total number of 70 patients who received 5-fluorouracil with or without cisplatin or mitomicin C and radiotherapy. The percentage of complete response in younger patients vs. women aged 65 and older were 78% and 66%, respectively. More severe complications were described in older patients, although not at a statistically relevant level [35].

At the time of writing, the most recent attempt to critically review the existing body of knowledge on the topic of interest seems to come from Gadducci and colleagues. Overall, the authors judge objective responses observed with neoadjuvant chemoradiation as satisfactory, with clinical complete response and pathological complete response ranging within the 25% to 72% and 31% to 52% intervals [36]. 

### 2.2. Primary Chemoradiation

The available studies of primary chemoradiation are listed in Table 2 [37,38,39,40,41,42,43,44,45,46]. Relevant aspects for the most representative trials are reported below. Evidence from the US National Cancer Database (NCDB), a clinical oncology database sourced from hospital registry data, showed that in 1352 patients treated with definite radiation or definite chemoradiation, 5-year overall survival was higher in the chemoradiation group (49.9% versus 27.4%, *p* < 0.001). This advantage was confirmed in the absence of node involvement, and independently from patients’ age [16]. A retrospective analysis on more than 2000 patients from the NCDB comparing definite radiotherapy or chemoradiation with the same treatment modalities with neoadjuvant intent showed similar survival rates for radiotherapy dosage greater than 55 Gy [47]. 

The Cochrane Systematic Review from Shylasree and collaborators was cited in the immediately preceding paragraph analyzed 141 patients included in three studies [29,30,43,44]. We now add on the two retrospective studies, which focused on primary chemoradiation versus primary surgery. Of note, in the first study, only toxicity was evaluated due to the particularly restricted number of patients treated [43,44]. Toxicity resulted considerable in patients treated with 5-fluorouracil and mitomicin C or mitomicin C alone [43]. Within the second retrospective study, weekly cisplatin or two cycles of cisplatin plus 5-fluorouracil was/were administered concurrently with radiotherapy vs. primary surgery. No significant difference in the risk of death emerged [44]. 

At present, chemoradiation is considered to be the standard of care in locally advanced vulvar carcinoma. Subsequent surgery is indicated in patients obtaining an objective response. Definite chemoradiation is applicable when the objective response is unsatisfactory, or in frail patients. A prospective phase II trial is ongoing, which evaluates cisplatin and gemcitabine in association with intensity modulated radiotherapy (IMRT) in the achievement of pathological complete response at subsequent surgery (NCT01595061) [48]. 

### 2.3. Neoadjuvant Chemotherapy

The exclusive use of chemotherapy in locally advanced vulvar cancer is mainly aimed at reducing surgery morbidity related to radiotherapy. The most representative literature on the use of neoadjuvant chemotherapy alone in locally advanced vulvar carcinoma is summarized in Table 3. Overall, the available studies are generally characterized by a restricted sample size, with the number of patients included varying within a 10 to 35 range. Remarkable heterogeneity in the chemotherapy regimens used is also a relevant issue. 

Shimizu and colleagues first reported on this topic in 1990. The administration of three cycles of bleomicin, vincristine, mitomycin C and cisplatin (BOMP) in a 57-year-old patient with inoperable vulvar cancer patient treated with BOMP resulted into a complete response and, subsequently, a radical surgery [49]. Encouraging evidence has come from a phase II EORTC study of 18 patients treated with bleomicin, methotrexate and lomustine. The reported response rate was 64% [50]. Benedetti–Panici and collaborators reported on the outcomes of 21 patients diagnosed with squamous cell vulvar carcinoma, who received neoadjuvant cisplatin, bleomicin and methotrexate. The neoadjuvant approach made surgery feasible in 19 patients (90.5%). A pathological complete response on inguinal nodes and in pelvic nodes was reported in 19% and 53% of patients, respectively. The 3-year overall survival was 24% [51]. According to the results of a further EORTC study on the combination of bleomycin, lomustine and methotrexate, 2 complete and 12 partial responses were observed among the 25 patients with locally advanced or recurred squamous cell vulvar carcinoma. Focusing on 12 patients with primary locally advanced disease, the overall response rate was set at 58% [52]. 

In the study carried out by Geisler and colleagues, 13 patients with tumors involving the urethra or anus were treated with neoadjuvant cisplatin plus 5-fluorouracil or platinum single agent. The use of the doublet resulted into a 100% objective response, with all of the patients undergoing radical surgery. Sphincters were preserved. Conversely, the exclusive use of cisplatin was not associated with any objective responses [53]. The outcomes of three different regimens of neoadjuvant treatment, i.e., bleomicin, paclitaxel and 5-fluorouracil + cisplatin, were evaluated in 25 patients with locally advanced disease. The use of bleomicin resulted into a 60% response rate, at the price of a higher toxicity and a 70% mortality rate. The overall response rates for the remaining regimens were 40% and 20%, respectively [54].

In a multicentric prospective study, 33 patients with locally advanced vulvar carcinoma were treated with four different cisplatin-based regimens or bleomicin as single agent. Among them, 27 (81.8%) underwent radical surgery [55].

Raspagliesi and collaborators reported on neoadjuvant paclitaxel + ifosfamide + paclitaxel or paclitaxel + cisplatin in a small case series including 10 patients. Subsequent surgery was performed in nine of these patients. Pathological complete response was exclusively reported in one patient, while carcinoma in situ was described in two patients [56].

### 2.4. Adjuvant Chemotherapy

To the best of our knowledge, only one study evaluated adjuvant cisplatin alone in 14 node-positive patients. Three-year progression free survival and overall survival were 71% and 86%, respectively [57].

### 2.5. Adjuvant Chemoradiation

Between 1984 and 1988, the outcome of chemoradiation with concurrent 5-fluorouracil with or without mitomicin C was explored in 33 vulvar cancer patients. Nine among them received adjuvant chemoradiation, and none had local recurrence, with seven patients being alive and disease free at a median follow up of 16 months [18]. 

In a further report, 6 out of 20 patients with vulvar cancer received adjuvant chemoradiation with 5-fluorouracil and mitomicin C. No significant advantage in survival was observed by chemoradiation compared with adjuvant radiation alone [15]. Forty-four patients with vulvar cancer treated with chemoradiation using concurrent platinum-based or 5-fluorouracil (this latter administered every 21/28 days), contributed data to the analysis from Mak and coauthors. Among them, 10 patients were treated in the adjuvant setting. No significant differences between the two chemotherapeutic regimens emerged concerning survival and disease free survival [45].

In the multicentric retrospective AGO-CaRE study, adjuvant radiotherapy or chemoradiation conferred a significant advantage in survival in node-positive patients, with 3-year progression free survival and overall survival being of 39.6% vs. 25.9% (*p* = 0.004), and 57.7% vs. 51.4% (*p* = 0.17), respectively. The addition of chemotherapy to radiation clearly offered benefit, reducing the risk of death with an HR of 0.62 [17]. 

## 3. Recurrent Disease

Multidisciplinarity is the hard core of patients’ management in recurrent disease. When disease recurs locally, it should be treated as a primary tumor whenever feasible. Viable options include surgery, radical radiation with/without chemotherapy, palliative radiotherapy, innovative therapies such as immunotherapy or targeted agents, palliative chemotherapy and supportive care. Unfortunately, the role of surgery in advanced or disseminated disease is extremely marginal. Palliative procedures may deeply impact quality of life, particularly in patients with pain and/or or in the case of fistulation of the tumors to bowel or bladder.

Palliative chemotherapy is a valid option for unfit patients, and for patients not amenable to further radiotherapy or surgery. Palliation of symptoms and improvement of the quality of life are the primary aims. Within this context, chemotherapy is chosen based on prior treatments, and often includes platinum derivatives, taxanes, pyrimidines, vinca alkaloids, gemcitabine and mitomycin C [58]. There is no available evidence orienting towards the use of one of these agents over another one, and there are no randomized studies. Responses are extremely uncommon, usually below 15% of the patients.

## 4. Advanced Disease

### 4.1. Chemotherapy, Chemoradiation and Electrochemotherapy

There is paucity of data on systemic treatment for vulvar carcinoma with extra pelvic disease. Unresectable or metastatic vulvar cancer has a relatively poor outcome despite the use of radiation therapy and chemotherapy, with a survival rate of approximately 20% at 5 years [15]. The low incidence of advanced vulvar carcinoma limits the feasibility of randomized clinical trials, with the treatments administered being more commonly extrapolated from cervical or anal carcinoma patients [59]. 

Along with the absence of standard treatment, key factors in decision making are represented by prior treatments, patient age and performance status. Unfortunately, vulvar cancer patients are, for the vast majority, relatively elderly and are therefore not optimal candidates for aggressive novel combinations. 

Anecdotal evidence is provided by numerous reports on single agents or drugs combinations [10]. Unfortunately in this case, the available literature is also quite dated, and mainly consists of retrospective studies including very limited patients’ numbers. In Table 4, we listed the available studies of chemotherapy alone in advanced vulvar cancer.

Platinum-based chemotherapy, especially in combination with paclitaxel, has become the standard of care in ovarian, endometrial and cervical cancers. Some experiences in vulvar cancer showed modest activity or anecdotal responses. Cisplatin as a single agent has showed response rate in less of 10% of the patients with advanced disease, suggesting an intrinsic chemoresistance of vulvar cancer [10]. The regimens most commonly used in advanced disease are paclitaxel, ciplatin/vinorelbine, cisplatin/gemcitabine or the doublet platinum-derivative/paclitaxel [60,61,62,63]. Other agents, such as mitoxantrone or bleomycin, did not yield any objective response [64,65].

In more detail, the combination of cisplatin and vinorelbine was evaluated in a small phase II trial including 15 chemotherapy-naive patients. The rate of response was 40%, including two complete responses; the median progression free survival was 10 months and the median overall survival 19 months. According to the authors’ conclusions, chemotherapy was well tolerated and showed evidence of efficacy in recurrent vulvar cancer [62]. In 29 patients participating in a phase II study of three-weekly paclitaxel as single agent in locally advanced, recurrent or metastatic vulvar cancer, the authors observed an overall response rate of 13.8% and a median progression free survival of 2.6 months, with grade 3–4 neutropenia in 27.6% of the patients, fatigue in 10.3% of the patients and neurotoxicity in 3.4% of the patients [60]. Evidence on the use of doublets platinum-based regimens (e.g., paclitaxel/carboplatin administered weekly) is from a small pilot study from Han and collaborators. Six patients with locally advanced or metastatic disease treated with the above regimen received a median number of 7.5 cycles, without any objective response and a discontinuation of the treatment after 1 month due to progressive disease in three out of six patients [63]. Chemoradiation may be an option even in advanced disease, and common current regimens include cisplatin-based chemoradiation, with or without 5-fluorouracil, or doublets with cisplatin or carboplatin in combination with a taxane [10].

A further treatment option is electrochemotherapy. An Italian retrospective case series has been recently published: 15 advanced patients were treated using bleomycin administered during the procedure. Overall, the 1 month response rate was 80%, with 8/13 patients alive at a 6-month follow-up [64]. 

Systemic treatment in later lines is mostly personalized. Particularly following first-line treatment with platinum-based combination regimens, objective responses to chemotherapeutic agents in subsequent lines are particularly discouraging, ranging among 10–15% [10,60]. The quality of the available evidence is quite low, mainly due to paucity of data and heterogeneity across the studies. At the time of this review writing and to the best of our knowledge, no guidelines have been established. Supportive care represents a backbone of disease treatment, aiming at improving quality of life [58].

### 4.2. Targeted Agents

Current research has focused on innovative treatment regimens, including biological agents and immunotherapy [10]. In order to personalize therapeutic options or adjunctive therapies for vulvar cancer, the exploration of the main molecular venues involved in the pathogenesis of vulvar carcinoma represents an active field of research, mainly focusing on aberrant cell cycle activity as a common pathway in all vulvar cancers, characterized by the overexpression of p53, Rb, cyclin D1, as well as the deregulation of EGFR expression or angiogenesis. Moreover, HPV-independent vulvar cancers are characterized by actionable mutations (including PIK3CA, CDKN2A and PTEN). Lastly, active research is ongoing on immunological targets, mainly in HPV-related vulvar cancers [66].

The role of tumor angiogenesis emerges through data on microvessel density, immunohistochemical evaluation, and from the high activity of antiangiogenetic agents observed in cervical cancer, in combination with chemotherapy [67]. The combination of newer agents such as paclitaxel and platinum with antiangiogenetics drugs has not been extensively studied in vulvar cancer yet. A recent case report on two patients with advanced vulvar cancer treated with paclitaxel/carboplatin/bevacizumab was published. A significant response on vulvar mass and on lung metastases was observed, and both patients continued maintenance bevacizumab [68]. The EGFR overexpression or gene amplification is frequently reported in vulvar carcinoma, and it is usually related to a worse prognosis [69]. The use of anti-EGFR agents, such as erlotinib, was tested in advanced vulvar cancer in a phase II study, with disappointing results, reporting only 27% of objective responses [70]. Recently, four patients with HER2 positive advanced vulvar Paget’s disease were treated with trastuzumab and paclitaxel, followed by maintenance trastuzumab. An objective response was observed in all the patients, with a median duration of response of 10 months. Treatment was extremely well tolerated [71].

Concerning potential novel targets, Kim and coauthors described a significant reduction of cell growth in a vulvar cancer cell line treated with celecoxib and cisplatin, in comparison with cisplatin alone, independently on Cox-2 expression in vulvar tissue [72]. Moreover, researchers from this same group showed the effect of an EGFR tyrosine kinase inhibitor (AG1478) alone and in combination with cisplatin on two vulvar cancer cells lines, and the growth inhibition was depending on EGFR expression, by inhibition of the activity of EGFR, AKT, ERK. The combination with cisplatin was not synergistic in either cell line [73]. 

In a case series recently published, targeted agents were given as second-third lines in cisplatin pretreated patients, reporting complete responses in two out of nine patients treated with bevacizumab, and partial responses in two out of five patients treated with erlotinib [74]. The use of immunotherapy, alone or in combination with chemotherapy, is an emerging and evolving field of research in vulvar carcinoma, opening a new scenario in this rare tumor, although the inherent data are still limited [75]. Concerning its use in combination with chemotherapy, the administration of pembrolizumab with weekly cisplatin and concurrent radiotherapy is being evaluated in a phase II study in unresectable or metastastic vulvar carcinoma [76].

## 5. Discussion

When the disease of interest falls within the “rare tumor” category, choosing the treatment that may better suit our patients’ needs is extremely challenging. Indeed, the impossibility of relying on high quality evidence from randomized trials is innately a severely limiting factor. As a matter of fact, vulvar carcinoma is a rare disease, representing the fourth most common gynecologic malignancy and 3–5% of all female genital tract tumors, with a 5-year survival rate of 19% for stage IVB patients [1,77,78].

Over the last 20 years, chemotherapy has been used in the treatment of vulvar cancer at multiple stages. In the neoadjuvant setting, chemotherapy is mainly delivered with the primary aim of reducing the extent of surgery, while in the adjuvant setting, mostly with concomitant radiation, for node positive disease, the main scope is reducing the risk of recurrence and maximize survival. Chemotherapy is also an option in recurrent and metastatic disease. In this latter context is increasingly used also in combination with targeted systemic therapies (e.g., epidermal growth factor receptor inhibitors, antiangiogenetics and immunotherapy).

The benefit of the exclusive use of chemotherapy in vulvar cancer has been initially explored in some small retrospective series, being vulvar cancer considered a chemoresistant tumor, and chemotherapy has been confined to the treatment of advanced stages, mostly as palliation [78]. Platinum-derivative, 5-fluorouracil, vinka alkaloids and taxanes, often given as doublet combination, are the chemotherapeutic agents most frequently used in vulvar carcinoma, and considered having some activity. Unfortunately, no significant advantages in terms of response rate and survival have been achieved with chemotherapy alone over the last twenty years in the advanced setting, with the 5-yr overall survival rates for stage IV being still set at 13% [79,80].

A case series from a phase II basket trial including one patient with recurrent squamous vulvar carcinoma and prior progression to systemic carboplatin showed a 30% reduction in PD-L1 positive tumor lesions following pembrolizumab [81]. More recently, pembrolizumab for MSI-high/dMMR or PD-L1 positive tumors has been suggested as second-line therapy, according to KEYNOTE-158 trial results [82]. Moreover, erlotinib has shown responses in one-fourth of a small cohort of patients with advanced disease [70], and bevacizumab offered promising results [68]. A phase I trial study is ongoing and evaluating the side effects and best dose of nelfinavir, an antiviral agent used in human immunodeficiency (HIV), when given together with cisplatin and external beam radiation therapy in treating patients with locally advanced vulvar cancer (ClinicalTrials.gov Identifier: NCT04169763). Lastly, Wang et al. tested the expression status of activated forms of CHK1 in 294 squamous vulvar carcinoma and correlated with clinical outcome. Moreover, CHK1 inhibition was tested in two vulvar cell lines, suggesting a role of CHK1 inhibitors in squamous vulvar carcinoma [83].

In conclusion, the systemic treatment of advanced vulvar cancer remains a challenge, since no new chemotherapeutic regimens or single agent showed significant activity, and in the last two decades there has been no significant improvement in outcome for advanced disease. To the best of our knowledge, preferred regimens in combination with radiation therapy are cisplatin as single agent or associated with 5-fluorouracil. The regimens most commonly employed in advanced or recurrent disease are platinum derivative, both as single agents or associated with taxanes. Other options are combinations of platinum derivative with vinorelbine or gemcitabine. Biological agents, given alone or in combination with chemotherapy, have shown limited activity until now. Promising ongoing trials focusing on immunotherapy are eagerly awaited. Deeper knowledge of targetable mutations and, more generally, on the biological pathway involved in vulvar cancer may help inform the use of innovative treatments based on targeted agents. In the meantime, a reduction in morbidity of chemoradiation and an improvement of patients’ quality of life remain of paramount importance.

## Figures and Tables

**Table 1:** Summary of studies with more than 10 patients exploring neoadjuvant chemoradiation in locally advanced vulvar cancer.

Study (First Author)	Number pts	Year	Chemotherapy Regimen	Radiotherapy Total Dose	Outcomes
Thomas et al. [18]	9 *	1989	5-FU 1000 mg/m^2^/d d1–4 with or without MMC 6 mg/m^2^	45–51 GY	cCR: 66.6%
Berek et al. [19]	12	1991	5-FU 1000 mg/m^2^ d1–4 + CisP 100 mg/m^2^ d1 for 2 cycles	40–52 Gy	cCR: 67%
Eifel et al. [20]	12	1995	CisP 4 mg/m^2^/d d1–4 + 5-FU 250 mg/m^2^/d d1–4 for 4 weeks	40 Gy	cPR: 100% pCR: 50%
Scheistroen et al. [21]	20	1993	Bleo 30 mg IV d1, 3, 5 for 2 courses	30–45 Gy	cCR: 25% cPR: 50%
Landoni et al. [22]	41	1996	5-FU 750 mg/m^2^ d1–5 + MMC 15 mg/m^2^ IV d1 for 2 courses	54 Gy in 2 courses	pCR: 24.4%
Lupi et al. [23]	24	1996	5-FU 750 mg/m^2^ d1–5 + MMC 15 mg/m^2^ IV d1 for 2 courses	54 Gy in 2 courses	cPR: 91.6%
Leiserowitz et al. [24]	23	1997	5-FU 1000 mg/m^2^ d1–4 + CisP 100 mg/m^2^ IV d2 for 2–3 cycles	54 Gy	cCR: 60.9%
Moore et al. [25]	71	1998	5-FU 1000 mg/m^2^ d1–4 + CisP 50 mg/m^2^ IV d1 for 2 courses	2 courses of 23.8 Gy	cCR: 48% pCR: 70%
Montana et al. [26]	46	2000	5-FU 1000 mg/m^2^ d1–4 + CisP 50 mg/m^2^ IV d1 for 2 courses	2 courses of 23.8 Gy	pCR (nodes): 40% pCR (vulva): 52%
Han et al. [27]	12	2000	5-FU 1000 mg/m^2^ d1–4 + MMC 10 mg/m^2^ IV d1 for 2 cycles	45 Gy	cCR: 71.4% cPR: 28.6%
Gerszten et al. [28]	18	2005	5-FU 1000 mg/m^2^ d1–4 + CisP 50 mg/m^2^ IV d1 for 2 courses	44.6 Gy	cCR: 72.2%
Gaudineau et al. [29]	22	2012	Carbo AUC 2 weekly	50 Gy	pCR: 27% pPR: 95%
Moore et al. [30]	58	2012	Weekly CisP 40 mg/m^2^ IV	57.6 Gy	cCR: 64% pCR: 50%
Beriwal et al. [31]	42	2013	CisP 40 mg/m^2^ d1 or 5-FU 1000 mg/m^2^ d1–5 for 2 courses	IMRT 46 Gy	cCR: 51.2% pCR: 48.5%

* 9/33: evidence is reported on 9 patients from a larger case-series of 33 patients. Abbreviations: 5-FU: 5-fluorouracil, AUC: area under the curve, Carbo: carboplatin, cCR clinical complete response, CisP: cisplatin, CR: complete response, d: days, Gy: Gray, IMRT: intensity-modulated radiation therapy, MMC: mitomycin C, ORR: overall response rate, pCR: pathologic complete response, PR: partial response, pts: patients.

**Table 2:** Summary of studies with more than 10 patients exploring primary chemoradiation in locally advanced vulvar cancer.

Study (First Author)	Number pts	Year	Chemotherapy Regimen	Radiotherapy Total Dose	Outcomes
Russel et al. [37]	18	1992	5-FU 750–1000 mg/m^2^ d1–4 + CisP 100 mg/m^2^ IV d1 for 2–3 cycles	54 Gy	CR: 50% PR: 6%
Koh et al. [38]	14	1993	5-FU 750–1000 mg/m^2^ IV d1–4 weekly	54 Gy	CR: 57% PR: 36%
Sebag-Montefiore et al. [39]	16	1994	5-FU 750 mg/m^2^ d1–5 + MMC 10 mg/m^2^ IV d1 for 2 cycles	45 Gy	CR: 44% PR: 37%
Wahlen et al. [40]	19	1995	5-FU 1000 mg/m^2^ d1–4 for 2 cycles with/without MMC 10 mg/m^2^ IV d1	45–50 Gy	CR: 52% PR: 36%
Cunningham et al. [41]	14	1997	5-FU 1000 mg/m^2^ d1–4 + CisP 50 mg/m^2^ d1 for 2 cycles	45–50 Gy	CR: 64% PR: 29%
Akl et al. [42]	12	2000	5-FU 1000 mg/m^2^/24h d1–4 and d29–32 + MMC 15 mg/m^2^ IV d1	30–36 Gy	CR: 100%
Mulayim et al. [43]	17	2004	5-FU 1000 mg/m^2^ d1–4 with/without MMC 10 mg/m^2^ IV d1	45–60 Gy	CR: 86%
Landrum et al. [44]	33	2008	Weekly CisP 40 mg/m^2^ or 2 cycles of CisP 50 mg/m^2^ IV d1 + 5-FU 1000 mg/m^2^ IV d1–4	47.6 Gy	CR: 87%
Mak et al. [45]	24	2011	weekly CisP or 5-FU based	50 Gy	CR: 58%
Tans et al. [46]	20	2011	5-FU 1000 mg/m^2^ infusion d1–4 + MMC 10 mg/m^2^ IV d1 for 2 courses	40 + 20 Gy	CR: 70%

Abbreviations: 5-FU: 5-fluorouracil, AUC: area under the curve, Carbo: carboplatin, CisP: cisplatin, CR: complete response, d: days, Gy: Gray, IMRT: intensity-modulated radiation therapy, MMC: mitomycin C, ORR: overall response rate, PR: partial response, pts: patients.

**Table 3:** Summary of studies with more than 10 patients exploring neoadjuvant chemotherapy in locally advanced vulvar cancer.

Study (First Author)	Number pts	Year	Chemotherapy Regimen	Outcomes
Durrant et al. [50]	18	1990	Bleo 5 mg IM d1–5 + MTX 15 mg PO d1 and 4 + CCNU 40 mg PO d5–7 week 1, then Bleo 5 mg IM d1 and 4 + MTX 15 mg PO d1 and 4	ORR: 64%
Benedetti-Panici et al. [51]	21	1993	CisP 100 mg/m^2^ d1 + Bleo 15 mg d1 and 8 + MTX 300 mg/m^2^ d8	ORR (vulva): 90.5% ORR (nodes): 67%
Wagenaar et al. [52]	12	2001	Week 1: Bleo 5 mg IM d1–5 + CCNU 40 mg PO d5–7 + MTX 10 mg PO d1 + 4 Weeks 2–6: Bleo 5 mg IM d1 + 4 + MTX 15 mg PO d1.	ORR: 58%
Geisler et al. [53]	13	2006	5-FU 1000 mg/m^2^/24 h d1–5 + CisP 50 mg/m^2^ IV d1 (*n* = 10)	pPR: 60%, pCR: 40%
			CisP 50 mg/m^2^ IV d1 (*n* = 3)	ORR: 0%
Domingues et al. [54]	25	2010	Bleo 20 mg/m^2^ IV d1–10 (*n* = 10)	ORR: 60%
			Tax 100 mg/m^2^ IV weekly (*n* = 5)	ORR: 40%
			5-FU 750 mg/m^2^ d1–4 + CisP 60–80 mg/m^2^ IV d1, weekly (*n* = 10)	ORR: 20%
Aragona et al. [55]	35	2012	Cis + 5-FU (*n* = 12) CisP + Tax (*n* = 6) CisP +5-FU + Tax (*n* = 6) VinC + Bleo + CisP (*n* = 6) Bleo alone (*n* = 5)	cPR: 85.7%
Raspagliesi et al. [56]	10	2014	Tax 175 mg/m^2^ d1 + ifosfamide 5 g/m^2^ in 24 h in d2 + CisP 50 mg/m^2^ Tax 175 mg/m2 d1 + CisP 70 mg/m^2^	pCR: 10%

Abbreviations: 5-FU: 5-fluorouracil, Bleo: bleomycin, CisP: cisplatin, CCNU: lomustine, pCR: pathological complete response, MTX: methotrexate, ORR: overall response rate, cPR: clinical partial response, pPR: pathological partial response, Tax: paclitaxel, VinC: vincristine.

**Table 4:** Summary of studies with more than 10 patients exploring chemotherapy alone in recurrent/metastatic vulvar cancer.

Study (First Author)	Number pts	Year	Chemotherapy Regimen	Outcomes
Thigpen et al. [61]	22	1986	CisP 50 mg/m^2^ IV q21 or Piperazinedione 9 mg/m^2^ IV q21	ORR: 0% mPFS: NA mOS: NA
Muss et al. [65]	11	1989	Mitoxantrone 12 mg/m^2^ IV q21	ORR: 0% mPFS: 1.3 m mOS: 3.2 m
Durrant et al. [50]	11	1990	Bleo 5 mg IM d1–5 + MTX 15 mg PO d1,4 + CCNU 40 mg PO d5–7 week 1 then Bleo 5 mg IM d1,4 + MTX 15 mg PO d1,4 weeks 2–5	ORR: 60% mPFS: NA mOS: NA
Wagenaar et al. [52]	13	2001	Week 1: Bleo 5 mg IM d1–5 + CCNU 40 mg PO d5–7 + MTX 10 mg PO d1,4 Weeks 2–6: Bleo 5 mg IM d1,4 + MTX 15 mg PO d1	ORR: 54% mPFS: 4.8 m mOS: 7.8 m
Cormio et al. [62]	15	2009	CisP 80 mg/m2 IV d1 + Vinorelbine 25 mg/m^2^ IV d1 and d8, q21	ORR: 40% mPFS: 10 m mOS: 19 m
Witteveen et al. [60]	29	2009	Tax 175 mg/m^2^ IV q3 weeks	ORR: 13.8% mPFS: 2.6 m mOS: 6.8 m

Abbreviations: Bleo bleomycin, CCNU: lomustine, CisP: cisplatin, NA: not available, ORR: overall response rate, m: months, mOS: median overall survival, mPFS: median progression-free survival, MTX: methotrexate, Tax: paclitaxel.
